# Supplementary material for: Effectors enabling adaptation to mitochondrial complex I loss in Hürthle cell carcinoma
Source: Cancer Discov. Author manuscript; Available in PMC 2023 Sep 27. (PMC10401073; doi:10.1158/2159-8290.CD-22-0976)
Supplement: 6 [file NIHMS1907325-supplement-6.pdf]

Supplementary Figures

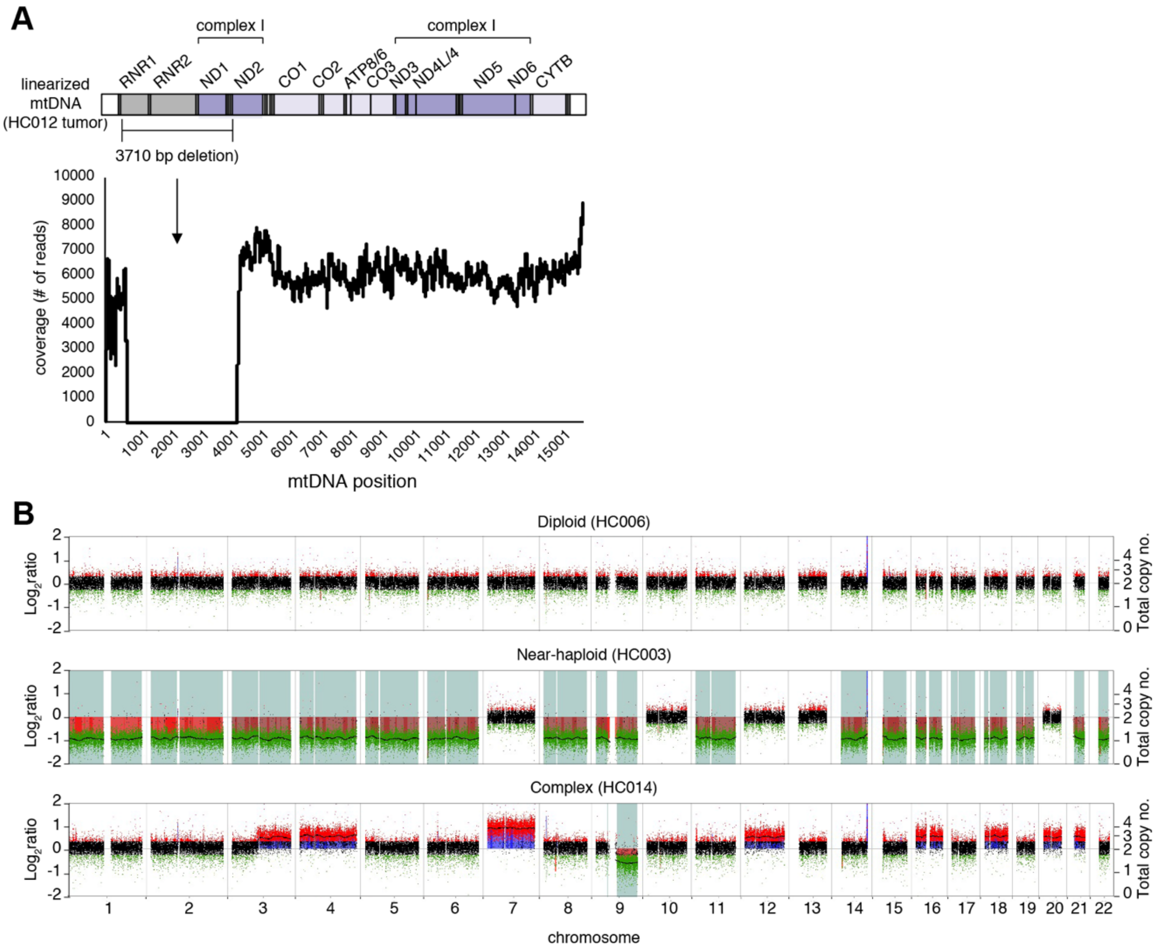

Supplementary Figure 1: Mitochondrial and nuclear DNA alterations in an HCC cohort. Related to Figure 1. (A) mtDNA coverage in tumor sample HC012 with location of 3710 bp shown on linearized mtDNA molecule with genes labelled. (B) Genomic plots showing copy number and LOH in HCC with illustrative diploid, near-haploid, and complex copy number profiles. Log_2 ratios (left y-axis) of the fluorescent intensities of the HCC sample vs a normal control were used to calculate copy number changes and to identify total copy number in tumor samples (right y-axis). Shaded areas indicate LOH.

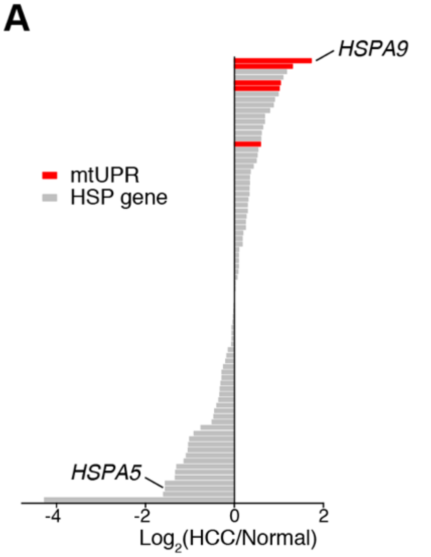

B

MITOCARTA PATHWAY	SIZE	NES	FWER p value
Protein Homeostasis	47	2.011993	0.005
Protein Import Sorting and Homeostasis	79	1.996833	0.005
Metals and Cofactors	110	1.980284	0.008
Mitochondrial Dynamics and Surveillance	91	1.970681	0.008
Apoptosis	33	1.937082	0.012

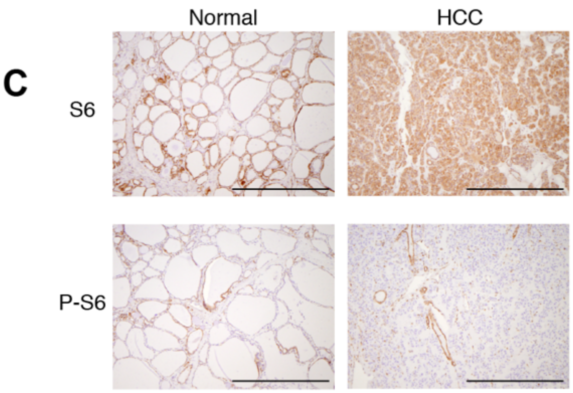

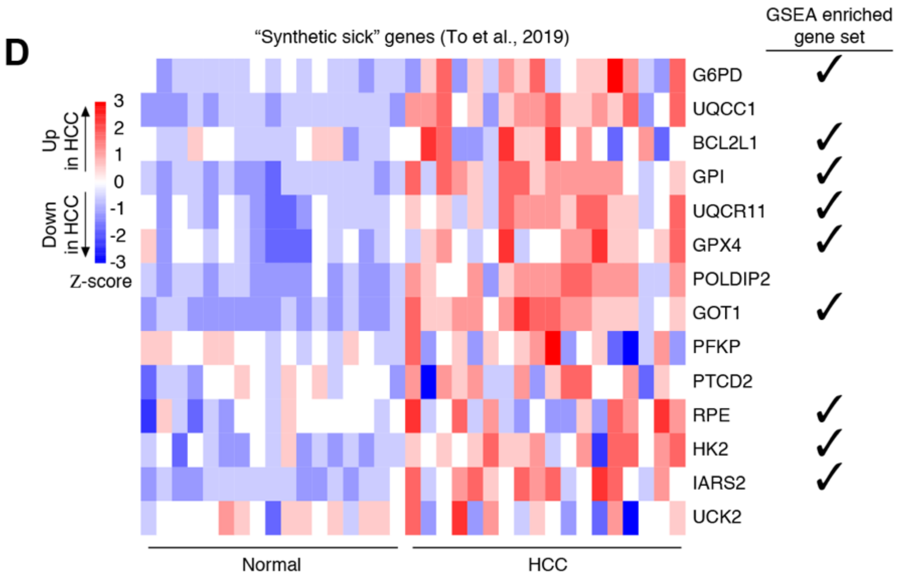

Supplementary Figure 2: Transcriptomic landscape of HCC. Related to Figure 2
(A) Gene expression fold-change of heat shock protein (HSP) members (Kampinga et al., 2008) in HCC with *HSPA9* (a mitochondrial HSP) and *HSPA5* (an endoplasmic reticulum HSP) highlighted. (B) Gene Set Enrichment Analysis (GSEA) of MitoCarta3.0 pathways (Rath et al., 2021) with FWER $p < 0.015$. (C) IHC for S6 and P-S6 (phospho-S6) proteins in HCC and normal thyroid; scale bar 800 μm. (D) Heatmap of genes whose knockouts were synthetic sick with OXPHOS dysfunction (To et al., 2019) with check marks indicating members of significantly enriched KEGG & HALLMARKS gene sets.

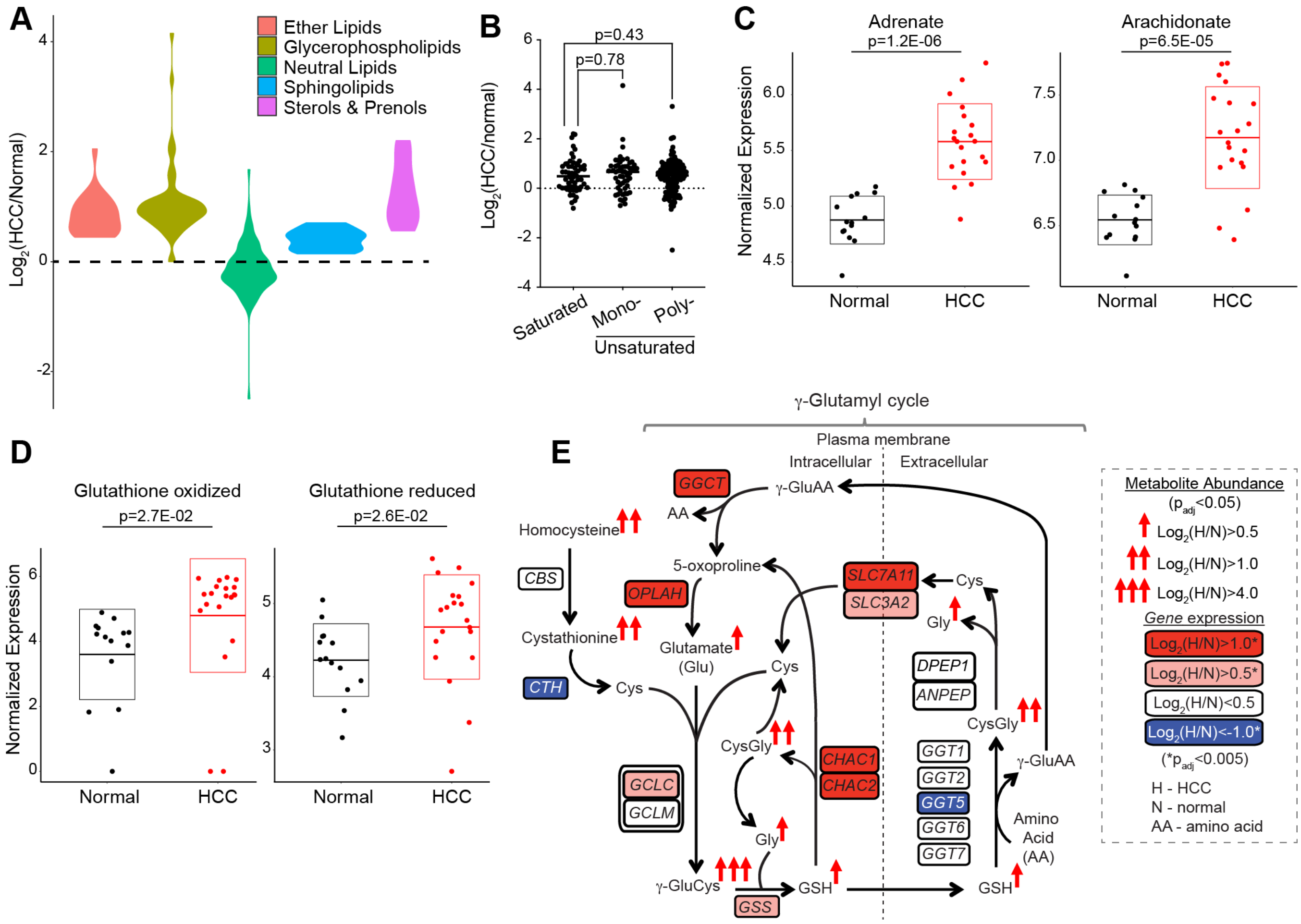

Supplementary Figure 3: Metabolic signatures of HCC. Related to Figure 3. (A) Violin plots of gene expression fold-changes grouped by lipid category. (B) Fold-change abundance of all classes of lipids in HCC according to saturation. Horizontal bars show median; p values from two-tailed t test. (C) and (D) Normalized expression of adrenate and arachidonate as well as oxidized and reduced glutathione with adjusted p values. Horizontal bars show mean and boxes standard deviation. (E) Schema of γ-glutamyl cycle with changes in gene expression and metabolite levels according to key.

A

HCC cell line karyotypes

MGH-HCC1: 49~53,XX,+7,+7,+12,+12,+20,+20,+21[cp20]

NCI-HCC: 70~77<3n>,XX,-Y,add(1)(q11)x2,del(3)(q26.3),+add(3)(p13),+add(3)(p21), add(4)(p16),-6,-6,-6,+7,-8,+i(10)(q10),-11,add(12(q22)x2,add(13)(p11.2), rob(13;15)(q10;q10),i(14)(q10)x2,+18,-19,+20,+20,-21,add(22)(q13)x2, del(22)(q12),+2mar1,+mar2,+4~6[cp5]

B

Cell line	complex I mtDNA variant	nuDNA SNaPshot
MGH-HCC1	*MT-ND1:* mG3745A	*CDKN2a -/-; PTEN -/-*
NCI-HCC	*MT-ND5:* mCA12417C	*TERT* C228T; *NF1* p.Arg1534Ter; *NF2* p.Gln362Ter; *CDKN2a -/-; PTEN -/-*
Nthy-ori 3-1	none	not tested

C

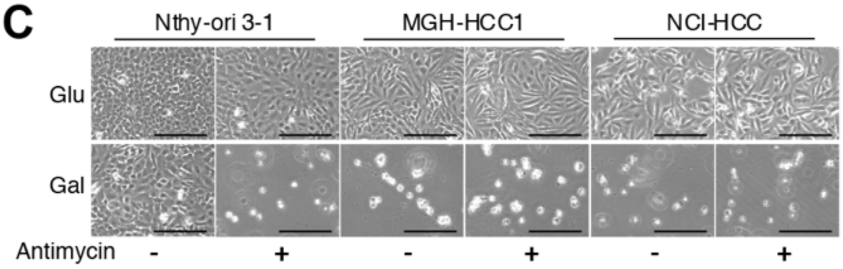

Supplementary Figure 4: Authentic models of HCC. Related to Figure 4. (A) Cytogenomic analysis of HCC cell line karyotypes based on GTG banding of metaphase spreads. (B) Table of mtDNA and nuclear DNA (nuDNA) variants. -/- indicates biallelic loss of ≥ 1 exon. In NCI-HCC cells there was homozygous loss of exon 1 in *CDK2NA* and homozygous loss of exons 2-8 in *PTEN*, while MGH-HCC1 had complete loss of both genes. (C) Brightfield images of cell lines in glucose or galactose ± antimycin; scale bar 250 μm.

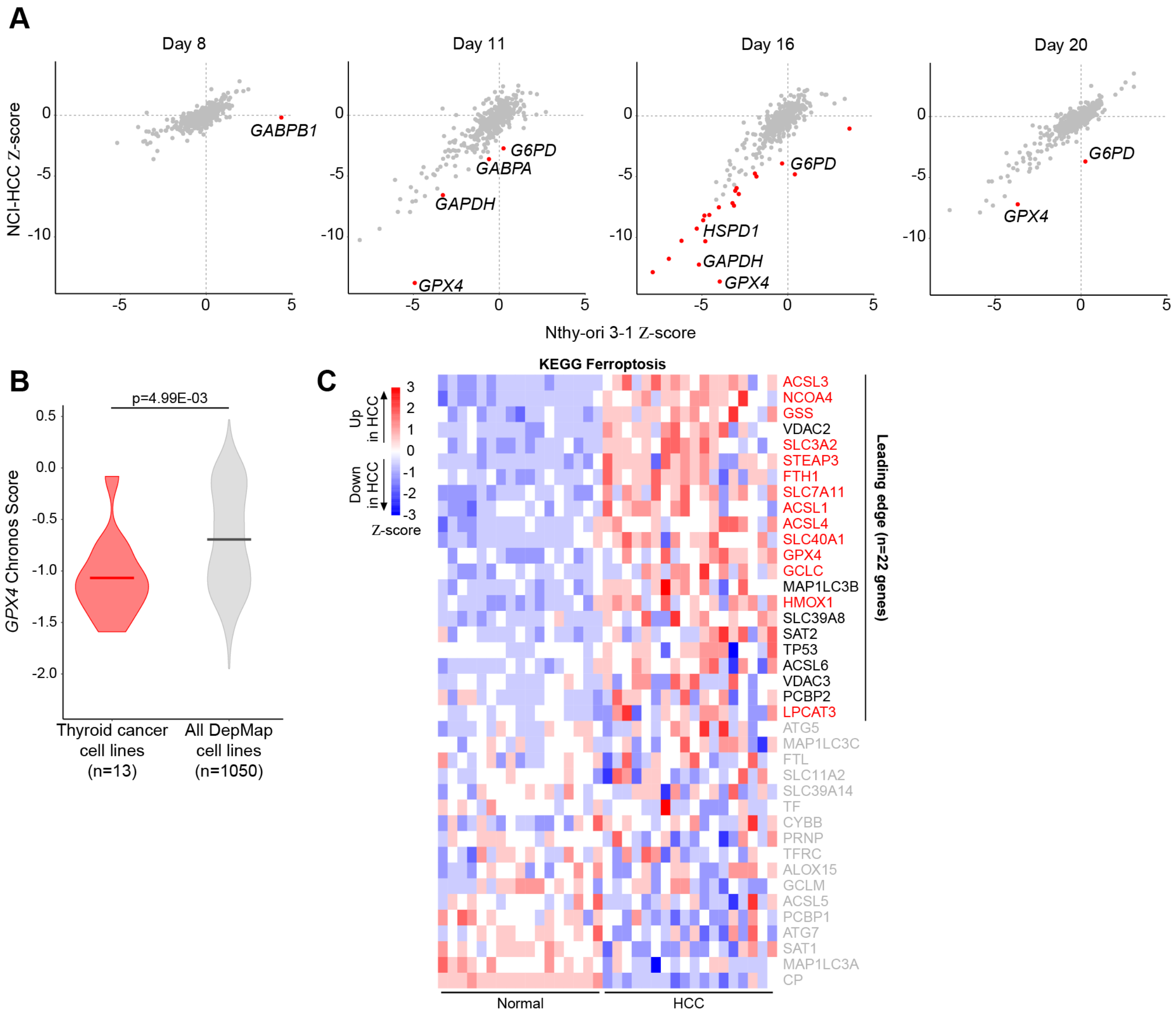

Supplementary Figure 5: CRISPR screen identifies vulnerability to GPX4 loss in HCC. Related to Figure 5. (A) Gene fitness scatter plots showing Z-scores in NCI-HCC (y-axis) vs Nthy-ori 3-1(x-axis) cells from Days 8, 11, 16 and 20. Red dots indicate genes with $\Delta Z < -2$ for a given day where $\Delta Z = (Z_{NCI\text{-}HCC} - Z_{Nthy\text{-}ori\ 3\text{-}1})/\sqrt{2}$ with the top 4 scoring genes labelled for Day 16. (B) Violin plots of *GPX4* chronos scores in thyroid compared to all other cancer cell lines. Horizontal lines show mean. (C) Ferroptosis heatmap with gene labels. Leading edge genes shown in red were used to create the metabolic schema in Figure 5D.

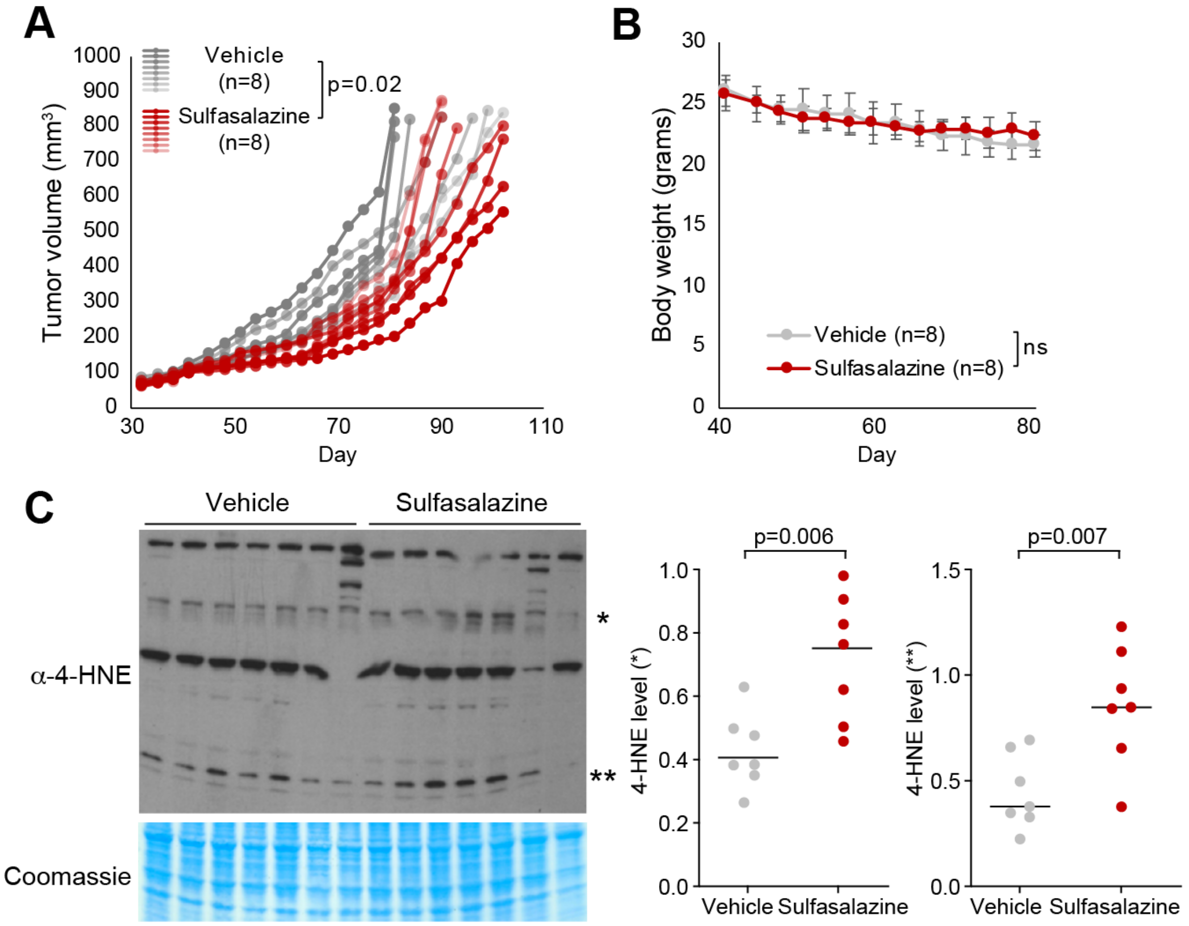

Supplementary Figure 6: NCI-HCC xenografts are sensitive to ferroptosis. Related to Figure 6. (A) Individual tumor volume (mm^3) growth curves of NCI-HCC xenografts treated with vehicle (5% DMSO) or 100 mg/kg sulfasalazine via daily IP injection (n=8 each). (B) Body weights of vehicle or sulfasalazine treated mice. (C) Western blot with 4-hydroxynonenal (4-HNE) antibody and Coomassie stained gel in vehicle and sulfasalazine treated NCI-HCC xenograft lysates (n=7 each). Laddering in lanes 7 and 13 likely due to sample degradation; * (~120 kDa) and ** (~30 kDa) represent regions of gel that were quantified; p values from unpaired t test; ns, $p > 0.05$.

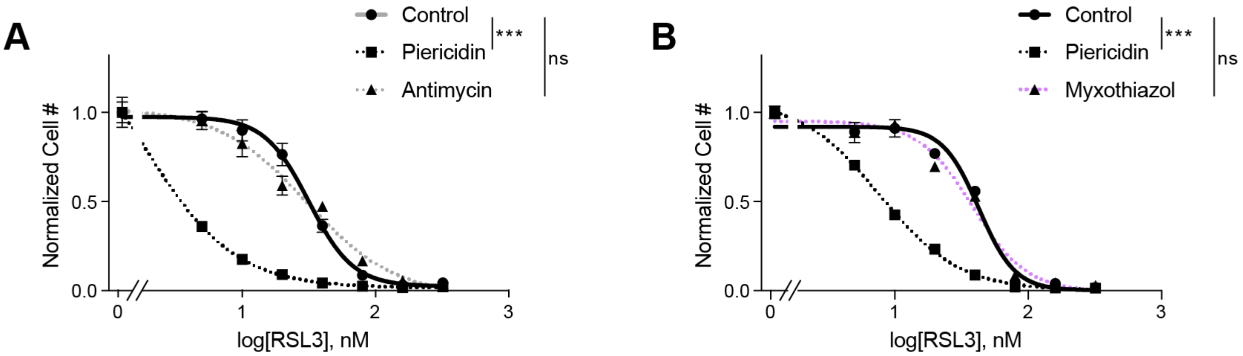

Supplementary Figure 7: Differential effects of OXPHOS inhibition on ferroptosis. Related to Figure 7. (A) Dose response curves in Nthy-ori 3-1 cells for RSL3 ± piericidin or antimycin plotted as normalized cell number; Mean ± SD; n=3, representative of two experiments. (B) Dose response curves in Nthy-ori 3-1 cells for RSL3 ± piericidin or myxothiazol plotted as normalized cell number; Mean ± SD; n=3, representative of two experiments. For (A) and (B), break in the x-axis is to allow visualization of 0 nM RSL3. Significance tested with extra sum-of-squares F test: ***$p < 0.0001$; ns, $p > 0.05$.
